# Matrine derivative YF-18 inhibits lung cancer cell proliferation and migration through down-regulating Skp2

**DOI:** 10.18632/oncotarget.14329

**Published:** 2016-12-28

**Authors:** Lichuan Wu, Guizhen Wang, Jinrui Wei, Na Huang, Sen Zhang, Fangfang Yang, Ming Li, Guangbiao Zhou, Lisheng Wang

**Affiliations:** ^1^ School of Chemistry and Chemical Engineering, Guangxi University, Nanning, Guangxi, PR China; ^2^ State Key Laboratory of Membrane Biology, Institute of Zoology, Chinese Academy of Sciences, Beijing, PR China; ^3^ Guangxi Scientific Research Center of Traditional Chinese Medicine, Guangxi University of Chinese Medicine, Nanning, Guangxi, PR China; ^4^ Affiliated Tumour Hospital of Guangxi Medical University, Nanning, Guangxi, PR China; ^5^ Key Laboratory of Animal Models and Human Disease Mechanisms of the Chinese Academy of Sciences and Yunnan Province, Kunming Institute of Zoology, Kunming, Yunnan, PR China

**Keywords:** lung cancer, matrine derivative, Skp2, proliferation, migration

## Abstract

Lung cancer is the leading cause of cancer related death which needs novel drugs to improve patient outcomes. In this study, we examined the ability of YF-18, a novel matrine derivative to inhibit the growth and migration of lung cancer cells. By cell cycle analysis, wound healing and transwell assays, we found that YF-18 induced G2/M cell cycle arrest and inhibited migration of lung cancer cells in a dose-dependent manner. Further results indicated that YF-18 inhibited cell proliferation and migration through down-regulating Skp2 and up-regulating its substrates, p27 and E-cadherin. Moreover, YF-18 inhibited A549-luciferase cell xenograft tumor growth in a dose-dependent manner. The findings indicate that YF-18 bears therapeutic potentials for lung cancer.

## INTRODUCTION

Lung cancer is the leading cause of cancer-related death, which has ranked first in men and second in women in morbidity and mortality [1]. Currently, chemotherapy is the main treatment for lung cancer, which has apparently reached a plateau of effectiveness in improving survival [2]. Although the survival of lung cancer patients has been improved with the emergence of tyrosine kinase inhibitors [3, 4], there still comes with new issues, such as drug resistance. It is still urgent to develop novel targeted drugs to improve patient outcomes.

Skp2 (S-phase kinase associated protein 2) is a component of the SCF E3 ubiquitin ligase (Skp, Cullin, F-box containing complex). Under normal physiological conditions, Skp2 controls cell cycle progression and cell survival by promoting the destruction of numerous proteins (Supplementary Table 1). Compared with normal tissues or cell lines, skp2 is found to be over-expressed in many different types of cancers [5–9], including lung cancer [10–14]. A growing body of evidence implicates that Skp2 promotes tumorigenesis [15–19]. Skp2 exerts its oncogenic functions via ubiquitination of tumor suppressor proteins, such as p27 [20] and E-cadherin [21]. Targeting Skp2 could induce cancer cell apoptosis and suppress tumorigenesis [22, 23], while inhibition of Skp2-mediated p27 and E-cadherin degradation leads to cell cycle blockage and migration inhibition [24, 25]. Collectively, these studies suggest that targeting Skp2 is a promising strategy for cancer therapy.

**Figure 1:**
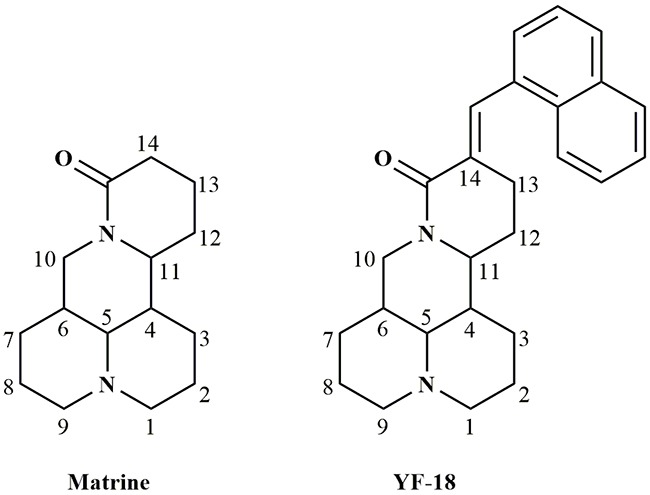
Structures of matrine and YF-18

Matrine (Figure 1) is the main chemical ingredient of Fufang Kushen injection which was approved by Chinese FDA (CFDA) in 1995. As an anticancer drug, Fufang Kushen injection could be used to treat non small cell lung cancer and liver cancer in combination with other anticancer drugs [26–31]. Owing to its druggable advantages, such as favorable solubility, flexibility structure and good safety profiles, matrine has been considered as an ideal lead compound for further modification [32–34]. In our previous study, we discovered that introduction of double bond and naphthalene ring at 14 site of matrine skeleton could largely increase its anticancer activity. In the current study, we designed and synthesized a new matrine derivative, YF-18 with a naphthalen-1-ylmethylene group modification (Figure 1). The mechanism of YF-18 on proliferation and migration inhibition was investigated.

## RESULTS

### YF-18 inhibits lung cancer cell proliferation, colony formation and arrests cell cycle at G2/M phase

**Figure 2:**
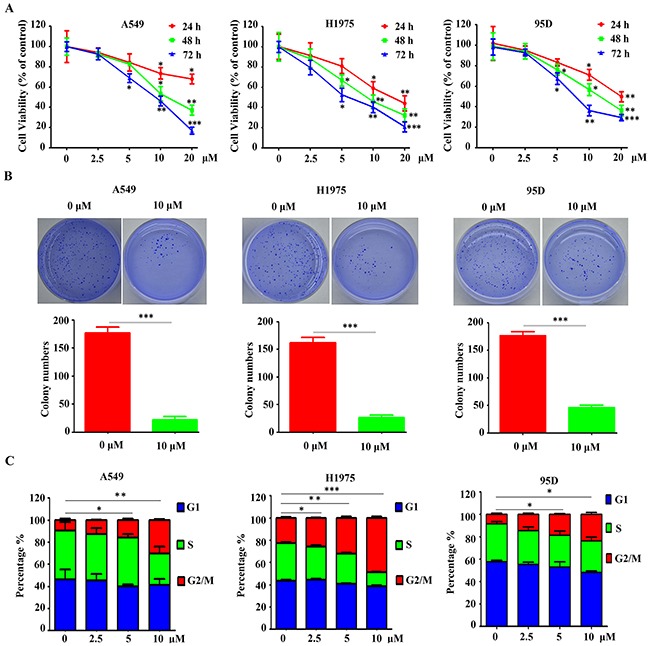
YF-18 inhibits lung cancer cell proliferation, colony formation and arrests cell cycle at G2/M phase **A**. A549, H1975 and 95D cells were treated with different concentrations of YF-18 for indicated time points and assessed by trypan blue exclusion analysis. **B**. Soft-agar colony formation assays for A549, H1975 and 95D cells treated with or without YF-18. **C**. YF-18 induced G2/M accumulation in a dose-dependent manner in A549, H1975 and 95D cells. Data is represented as mean ± SD.

The synthesis and characterization of YF-18 was described in supplementary information. To detect whether YF-18 treatment inhibits cell growth in lung cancer cells, MTT assays were performed in A549, H1975 and 95D cells treated with different concentrations of YF-18 for indicated times. The results showed that YF-18 potently inhibited cell growth in a time- and dose-dependent manner (Figure 2A). The IC_50_ at 48 hours for A549, H1975 and 95D was 15 μM, 10 μM and 12 μM, respectively. Therefore, we used dose of 10 μM in the following studies. Moreover, the colony formation results demonstrated that YF-18 significantly inhibited lung cancer cell colony forming activity (Figure 2B). To further define the proliferation inhibition by YF-18 on lung cancer cells, we conducted cell cycle analysis and cell apoptosis assay in A549, H1975 and 95D cells. Cells were treated with different concentrations of YF-18 for 24 hours and analyzed by FACS. The cell cycle analysis results showed that YF-18 arrested cell cycle at G2/M phase in A549, H1975 and 95D cells in a dose-dependent manner. The percentage of G2/M phase in control of A549, H1975 and 95D cells was around 10%, 22% and 9%. With the treatment of YF-18 (10 μM), the percentage of G2/M phase increased to 30%, 48% and 24%, respectively (Figure 2C). The cell apoptosis results revealed that YF-18 didn't induce cell apoptosis in A549, H1975 and 95D cells (Supplementary Figure 1). Collectively, it can be inferred that YF-18 inhibited cell proliferation through cell cycle arrest not cell death.

### YF-18 inhibits cell migration in lung cancer cells

**Figure 3:**
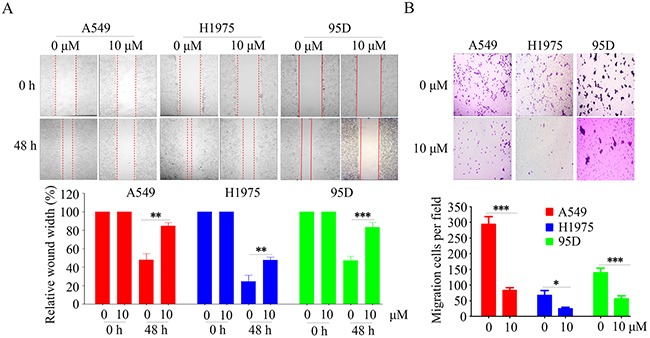
YF-18 suppresses cell migration in lung cancer cells **A**. Inhibition of cell migration by YF-18 was confirmed by the wounding healing assay. **B**. Treatment with YF-18 reduced the number of migrated cells in the transwell assay. Data is represented as mean ± SD.

To analyze whether YF-18 could inhibit the motility of lung cancer cells, we performed wound healing and transwell assays. The wound healing results demonstrated that YF-18 significantly decreased cell migration in A549, H1975 and 95D cells (Figure 3A). Consistently, YF-18 treatment led to decreased penetration of lung cancer cells from transwell assay (Figure 3B).

### YF-18 down-regulates Skp2 and up-regulates p27 and E-cadherin

**Figure 4:**
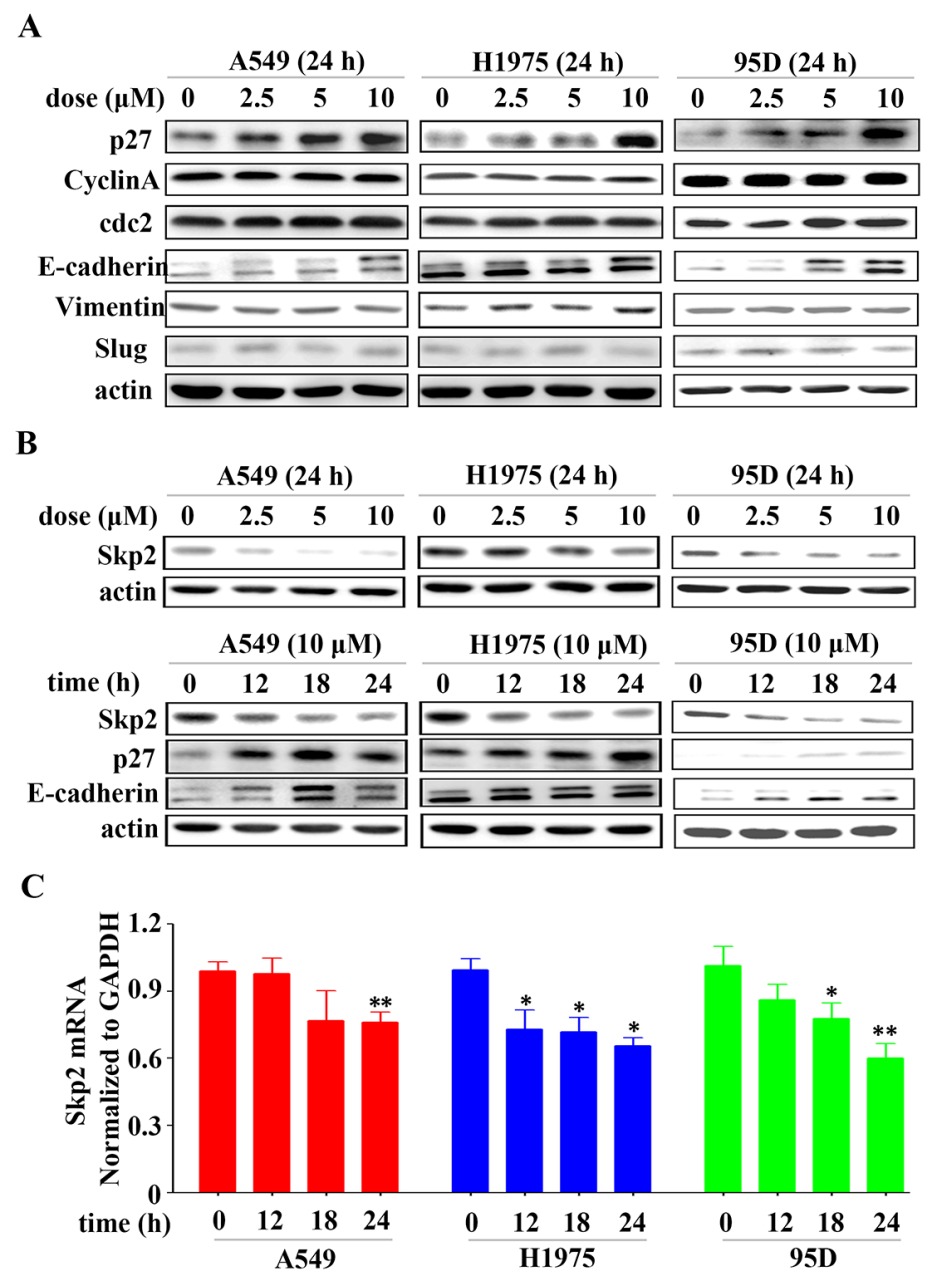
YF-18 treatment leads to Skp2 down-regulation and its targets p27 and E-cadherin up-regulation **A**. P27 and E-cadherin were up-regulated upon YF-18 treatment. **B**. YF-18 reduced Skp2 expression in a time- and dose-dependent manner. **C**. Treatment with YF-18 led to transcription inhibition of Skp2. Data is represented as mean ± SD.

To dissect the molecule mechanism underlying YF-18 induced G2/M cell cycle arrest and migration inhibition, we performed western blot assays to detect regulators involved in G2/M and cell migration. The results demonstrated that p27 and E-cadherin were dramatically increased upon YF-18 treatment in a dose-dependent manner (Figure 4A). Accumulative evidence demonstrates that Skp2 could mediate p27 and E-cadherin degradation [35]. By western blotting and real time PCR assays, we found that YF-18 down-regulated Skp2 at both the protein and mRNA levels in a dose- and time-dependent manner in A549, H1975 and 95D lung cancer cells (Figure 4B and 4C). Consistently, p27 and E-cadherin were up-regulated in a time-dependent manner (Figure 4B). Collectively, these results implied that YF-18 could increase p27 and E-cadherin expression through decreasing Skp2 expression.

### YF-18 inhibits lung cancer cell proliferation and migration through down-regulating Skp2

**Figure 5:**
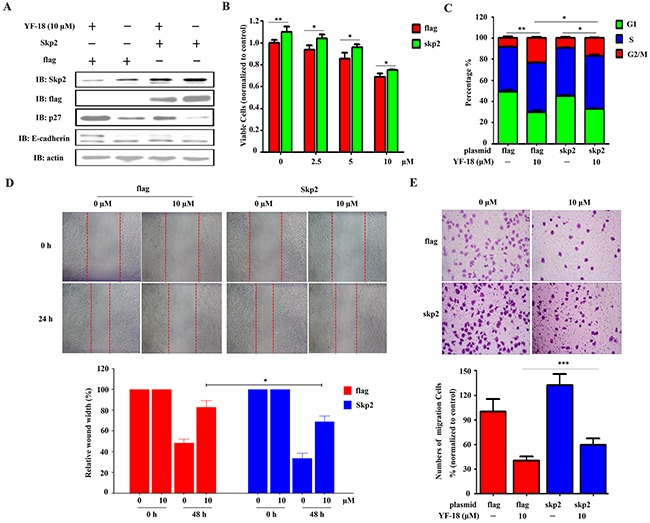
Over-expression of Skp2 attenuates YF-18 induced proliferation inhibition, G2/M phase accumulation and migration suppression **A**. A549-luciferase cells were transfected with Skp2 and treated with different concentrations of YF-18 for 24 hours. Western blot assays were involved to detect the expression of skp2, p27, and E-cadherin. **B**. A549-luciferase cells were transfected with Skp2 and treated with YF-18. Viable cells were detected by MTT assay. **C**. A549-luciferase cells were transfected with Skp2 and treated with YF-18 for 24 hours. The cells were analyzed by flow cytometry to evaluate the cell cycle distribution. **D and E**. A549-luciferase cells were transfected with Skp2 and wound healing (D) and transwell (E) assays were conducted to evaluate cell migration. Data is represented as mean ± SD.

To elucidate whether YF-18 exerts its anticancer activity through down-regulating Skp2 in lung cancer cells, A549-luciferase cells were transfected with Skp2 plasmid or empty vector as control and treated with YF-18 or not for further experiments. The expression of Skp2, p27 and E-cadherin was detected by western blotting (Figure 5A). Subsequently, MTT, cell cycle, wound healing, and transwell assays were conducted. We found that over-expression of Skp2 could promote cell growth, and also attenuate cell proliferation inhibition by YF-18 treatment (Figure 5B). The cell cycle analysis results showed that over-expression of Skp2 inversed YF-18-induced G2/M arrest (Figure 5C). Upon YF-18 treatment, the percentage of cells at G2/M phase transfected with empty vector rose from 8% to 23%, while the percentage of cells at G2/M phase transfected with Skp2 plasmid rose from 9% to 17%. Skp2 over-expression decreased G2/M accumulation induced by YF-18 with about 7%. Also, the wound healing and transwell assays results demonstrated that over-expression of Skp2 increased lung cancer cell migration in both YF-18 treatment and vehicle control and reversed cell migration inhibition by YF-18 treatment (Figure 5D and 5E). Collectively, these results suggested that YF-18 inhibited lung cancer cell proliferation and migration through down-regulating Skp2.

### *In vivo* anti-lung cancer activity of YF-18

**Figure 6:**
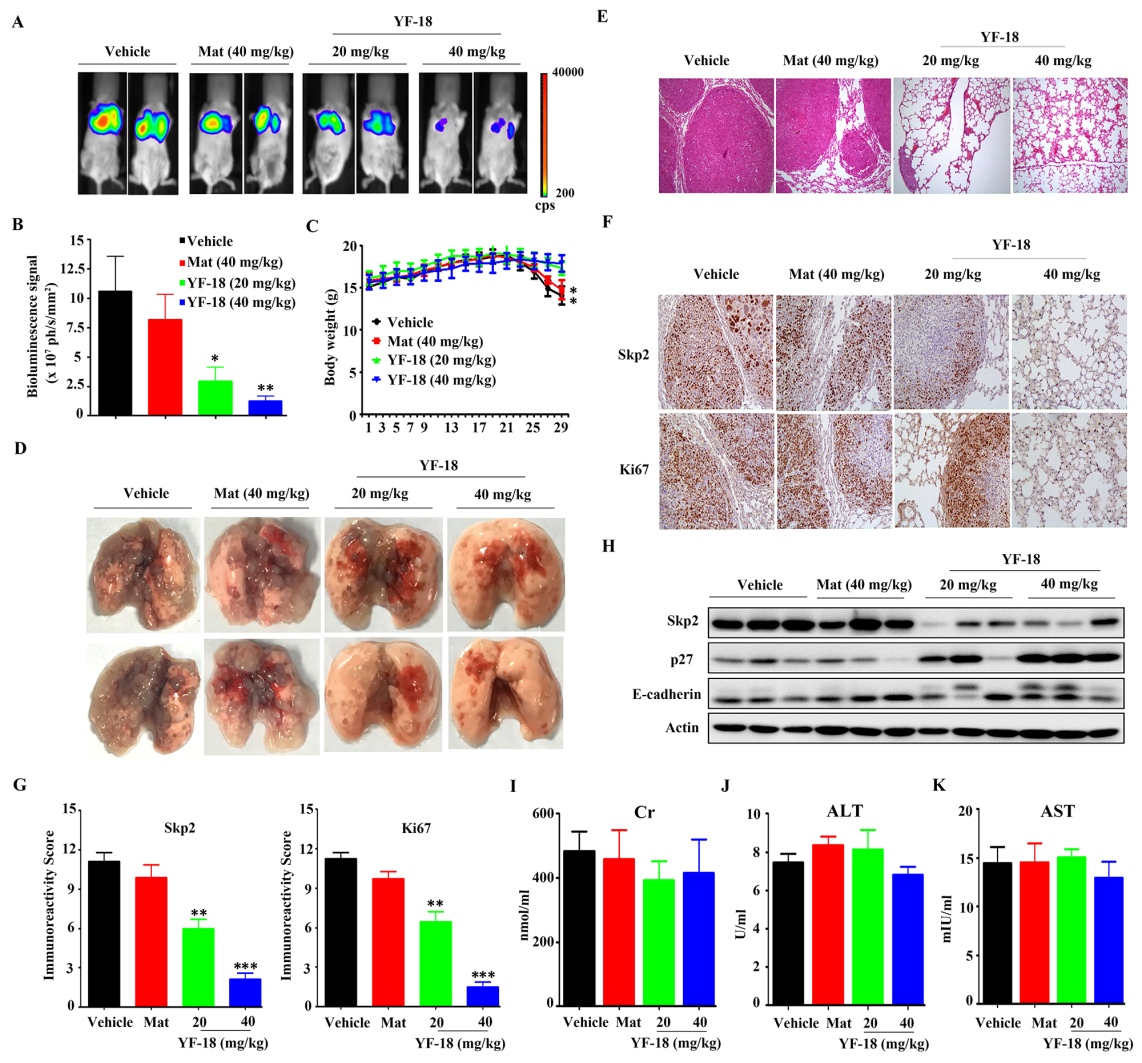
*In vivo* anti-lung cancer efficacy of YF-18 **A**. A549-Luciferase cells were intravenously injected into SCID mice, and 4 days later the mice were randomized to receive vehicle, matrine, and YF-18 treatment (*n* = 6 for each group). The mice were detected by IVIS Spectrum. **B**. The relative luciferase intensity in the mice. **C**. The body weight of mice was monitored every two days. **D**. Representative images of dissected lung tissue from each group. **E**. Hematoxylin and eosin (HE) staining of lung tissue sections of mice from each group. **F**. Immunohistochemistry (IHC) using anti-Skp2 and Ki67 antibodies. **G**. Statistic analysis of IHC staining. **H**. Western blot assays using lysates of isolated tumors and indicated antibodies. **I-K**. The serum Cr (I), ALT (J) and AST (K) levels of mice from each group were detected. Data is represented as mean ± SD.

To evaluate the anti-lung cancer activity of YF-18 *in* *vivo*, A549-luciferase cells (1×10^6^) were intravenously injected into SCID/Beige mice (*n*=6 for each group). Vehicle, YF-18 (20, 40 mg/kg), and matrine (40 mg/kg) were intraperitoneally administrated every other day for 3 weeks. The results demonstrated that YF-18 significantly suppressed tumor growth reflected by decrease of luciferase bioluminescence intensity, while matrine had no obvious effect (Figure 6A and 6B). YF-18 treatments did not lead to body weight reduction while in vehicle and matrine groups, the body weight loss began at day 23 dramatically (Figure 6C). Tumor can be obviously found in the dissected lung tissue of vehicle and matrine group, while in YF-18 (40 mg/kg) group, tumor size decreased dramatically (Figure 6D). Consistent with the results in Figure 6D, YF-18 reduced dissemination of disease and prevented destruction of tissue architectures reflected by HE staining (Figure 6E). Immunohistochemistry analysis results indicated that YF-18 induced down-regulation of Skp2 in tumor samples (Figure 6F–6G). Western blotting results revealed that YF-18 down-regulated Skp2 and up-regulated p27 and E-cadherin *in vivo* (Figure 6H). We also tested the adverse effects of YF-18. Compared with the vehicle control, the results demonstrated that mice treated with YF-18 had normal serum concentration of creatinine (CR), Alanine Aminotransferase (ALT) and Aspartate aminotransferase (AST) (Figure 6I–6K). These results inferred that YF-18 displayed favorable anti-tumor effect *in vivo* with no obvious side effects.

## DISCUSSION

Matrine, the main component of *Sophora flavescens Ait* which is listed in Chinese Pharmacopoeia, has been approved as an adjuvant drug for preventing cachexia in China. Clinical studies have demonstrated that the quality of life and immune function of cancer patients were largely improved by combining standard therapies with the use of matrine [36, 37]. Besides, it is well documented that matrine can inhibit proliferation of a variety of cancer cells [38–40]. Therefore, matrine could be an ideal lead compound for anticancer drug discovery. In this study, we synthesized a new matrine derivative YF-18, and found that YF-18 could potently inhibit lung cancer cell proliferation *in vitro* and *in vivo* (Figure 2, Figure 6A-6B, 6D-6F). Interestingly, YF-18 exhibits slight side effect reflected by body weight loss and ALT, AST and Cr detection (Figure 6C and 6I-6K). These results implied that YF-18 displayed advantage in drug safety and druggable potential.

Since Skp2 is firstly reported to be over-expressed in lung cancer [41], more and more studies have been involved to elucidate the role of Skp2 in lung cancer progression. It is reported that Skp2 plays an oncogenic role in the pathogenesis of lung cancer [11, 42–44]. High expression of Skp2 is clinically correlated with low expression of p27, a tumor suppressor and critical regulator of cell cycle. In this study, we found that YF-18 induced G2/M arrest was correlated with p27 up-regulation via down-regulating Skp2 (Figure 2C, Figure 4A-4B, Figure 5A, 5C). E-cadherin, which is a key regulator mediating cell migration, is observed with a low expression in lung cancer and negatively regulated by Skp2 [21]. Loss of E-cadherin is considered as a hallmarker of epithelial-mesenchymal transition (EMT) [45]. In the early stagy of EMT, cells expressed decreased E-cadherin and acquired enhanced migration ability [46]. Thus, restoring E-cadherin expression can inhibit cell migration and prevent EMT initiation. In our study, we discovered that YF-18 could restore E-cadherin expression and inhibit migration (Figure 3 and Figure 4A-4B). Further results indicated that YF-18 induced migration inhibition was correlated with down-regulation of Skp2 (Figure 5A, 5D and 5E). Considering the role of E-cadherin and migration on EMT, we speculate that YF-18 might inhibit lung cancer cell metastasis through regulating EMT process. Certainly, for future follow ups, we would test the effects of YF-18 on lung cancer metastasis *in vivo* and elucidate the role of EMT in YF-18 induced metastasis inhibition.

In summary, we conclude that YF-18 inhibits cell growth, induces G2/M cell cycle arrest, and suppresses cell migration via down-regulating Skp2 in lung cancer cells. Our results suggest that inhibiting Skp2 by YF-18 could be a potential effective approach to treat lung cancer.

## MATERIALS AND METHODS

### Cell culture, cell proliferation and cell viability assays

The lung cancer cell lines A549 and H1975 were purchased from ATCC (American Type Culture Collection) and cultured as described [47, 48]. Highly metastatic large cell lung cancer line 95D was obtained from the Cell bank of Chinese Academy of Sciences (Shanghai, China). The cells (5000 cells per well) were plated in flat-bottomed 96-well micro plates. Sixteen hours after seeding, new medium containing different concentrations of YF-18 or solvent control (DMSO) was added. Cells were further incubated for indicated times and incubated with MTT for additional 2-4 hours. The plates were then assayed by testing the absorbance at 490 nm. Cell viability was estimated by trypan blue dye exclusion assay [49].

### Clonogenic assay, cell cycle and apoptosis analysis

For clonogenic assay, cells were suspended in 1 ml DMEM containing 0.3% low-melting-point agarose (Amresco, Solon, OH) and 10% FBS, and plated on a bottom layer containing 0.6% agarose and 10 μM YF-18 in 35 mm plates (1000 cells/plate). After 14 days of culture, cells were stained with Giemsa and clones containing more than 50 cells were counted. For cell cycle analysis, cells were treated with YF-18 at different concentrations for 24 hours, and DNA content was determined by PI staining and flow cytometry analysis. For cell apoptosis, cells were treated with YF-18 at 10 μM for 24 hours. Externalization of phosphatidylserine was tested using an Annexin V/PI Apoptosis Detection kit (BD Biosciences, San Jose, CA) according to manufacturer's instruction.

### Wound-healing assay and *in vitro* migration assay

For wound-healing assay, cells (5 × 10^5^/well) were seeded into six-well plates. Twenty-four hours later, wounds were created in the cell monolayer using a white micropipette tip. Fresh medium containing 10 μM YF-18 was added and the healing process was followed for the next 48 hours. For the transwell assay, transwell inserts (6.5 mm diameter and 8 μm pore size; Corning Inc., Corning, NY, USA) were rehydrated in advance by adding serum-free medium for at least 1 hour. Cells in serum-free medium were seeded (2 × 10^4^ cells) into the inserts (the upper chamber). Complete medium containing serum was used as a chemoattractant in the bottom chamber, and 10 μM YF-18 was added to inhibit cell migration. After 24 hours of migration, cells in the upper surface of the insert membrane were removed by wiping with a cotton swab, and cells in the lower surface were fixed with methanol, stained with crystal violet, and counted by microscopy.

### Western blotting and quantitative RT-PCR assays

For western blotting assay, cells were lysed in RIPA buffer supplemented with protease inhibitors. Proteins (20 μg) were subjected to 6–15% SDS-PAGE, electrophoresed and transferred on to a nitrocellulose membrane. After blocking with 5% non-fat milk in Tris-buffered saline, the membrane was washed and incubated with the indicated primary and secondary antibodies and detected using the Luminescent Image Analyser LSA 4000 (GE, Fairfield, CO, USA). To detect the mRNA expression of related genes, qPCR was conducted with SYBR™ Green Real time PCR Master Mix (Takara Biotechnology, Dalian, China). The primers used for quantitative RT-PCR are as follows: Skp2, forward, 5’-GCTGCTAAAGGTCTCTGGTGT-3’ and reverse, 5’-AGGCTTAGATTCTGCAACTTG-3’, GAPDH, forward, 5’-GAGTCAACGGATTTGGTCGT-3’ and reverse, 5’-GACAAGCTTCCCGTTCTCAG-3’.

### Plasmids and transfection

The coding sequence of Skp2 was cloned into the pcDNA3.1-flag expression vector (Invitrogen, Carlsbad, CA, USA). Using lipofectamine 2000 (Invitrogen, California, USA), A549-luciferase cells were transfected with 1 μg plasmids. And 48 hours later, the cells were treated with or without YF-18 at 10 μM for indicated time points. The cells were then harvested for cell proliferation, cell cycle analysis, wound healing and transwell assays or lysed for Western blotting to detect the expression of Skp2, p27 and E-cadherin.

### Immunohistochemistry analysis

IHC assay was performed using anti-Skp2 and anti-Ki67 antibodies as previously described [50]. Briefly, formalin-fixed, paraffin-embedded mouse lung cancer tissue specimens (5 mm) were deparaffinized through xylene and graded alcohol, and subjected to a heat-induced epitope retrieval step in citrate buffer solution. The sections were then blocked with 5% BSA for 30 min and incubated with indicated antibodies at 4 °C overnight, followed by incubation with secondary antibodies for 90 min at 37 °C. Detection was achieved with 3, 3’-diaminobenzidine (DAB, Zhongshan Golden Bridge Biotechnology, Beijing, China) and counterstained with hematoxylin, dehydrated, cleared and mounted as in routine processing.

### Murine models

The animal studies were approved by the Institutional Review Board of Institute of Zoology, Chinese Academy of Sciences. All animal studies were conducted according to protocols approved by the Animal Ethics Committee of the Institute of Zoology, Chinese Academy of Sciences. SCID/beige mice were injected with A549-luciferase (A549-Luc) cells (1 × 10^6^) via tail vein, and 4 days later the mice were randomized into 4 groups to receive treatment with intraperitoneal injection of vehicle, matrine at 40 mg/kg, YF-18 at 20 and 40 mg/kg (*n*=6 for each group; once every two days for 21 days). The mice were imaged by IVIS Spectrum at day 21, and were euthanized by cervical dislocation at day 30.

### Statistical analysis

The data are presented as the mean ± SD. Differences between data groups were evaluated for significance using Student's t-test of unpaired data. P values < 0.05 were considered statistically significant.

## SUPPLEMENTARY MATERIALS FIGURE AND TABLE



## Abbreviations

MTT: Methylthiazolyldiphenyl-tetrazolium bromide
FACS: Fluorescence Activated Cell Sorter
SCF complex: Skp, Cullin, F-box containing complex
