# Supplementary material for: Matrine derivative YF-18 inhibits lung cancer cell proliferation and migration through down-regulating Skp2
Source: Oncotarget. 2016 Dec 28;8(7):11729–38. doi: 10.18632/oncotarget.14329 (PMC5355299; doi:10.18632/oncotarget.14329)
Supplement: Supplementary file 1 [file oncotarget-08-11729-s001.pdf]

Matrine derivative YF-18 inhibits lung cancer cell proliferation and migration through down-regulating Skp2

SUPPLEMENTARY MATERIALS

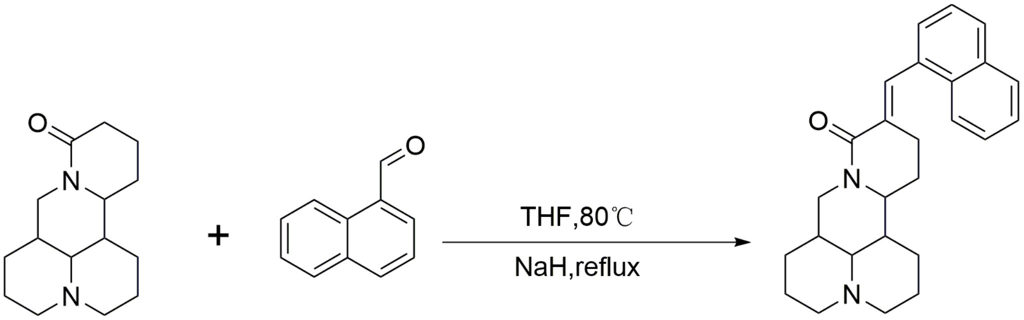

Scheme 1: Synthetic route for compound YF-18.

Synthesis and characterization of YF-18

Sodium hydride 2.8 g (116 mmol) was added into 50.0 ml anhydrous THF and then matrine 1.24g (5 mmol) was added. The reaction mixture was stirred with slow warming to 80 °C for 30min. 1-Naphthaldehyde 1.56 g (10 mmol) was added into the mixture. The reaction termination was monitored by TLC, then cooled down to room temperature and the solution was acidified with 3N HCl until pH reached to 7-8. The aqueous phase was extracted with dichloromethane (30 x 3), and the combined organic phases was washed with water, brine, and dried (Na_2SO_4) sequentially, then filtered and evaporated under reduced pressure to give a yellow residue. Finally, the yellow residue was purified with silica gel column chromatography (petroleum ether/ethyl acetate = 1:1) to give compound YF-18.

Characterization of YF-18

White solid (49% yield), m.p. 173.5-176.3 °C; ^{1}H NMR (600 MHz, CD_3Cl): δ ppm 8.22 (s, 1H), 8.04~8.00 (m, 1H), 7.90~7.87 (m, 1H), 7.83 (d, J = 8.4 Hz, 1H), 7.54~7.51 (m, 2H), 7. 48 (t, J = 7.8 Hz, 1H), 7.33 (d, J = 7.2 Hz, 1H), 4.59 (dd, J = 4.2 Hz, J = 12.6 Hz, 1H), 4.02~3.97 (m, 1H), 3.28 (t, J = 12.6 Hz, 1H), 2.92~2.84 (m, 2H), 2.73~2.68 (m, 1H), 2.42~2.35 (m, 1H), 2.19 (s, 1H), 2.12~2.05 (m, 1H), 1.92~1.76 (m, 3H), 1.71~1.57 (m, 4H), 1.53~1.42 (m, 4H), 1.36~1.26 (m, 2H); ^{13}C NMR (150 MHz, CD_3Cl): δ ppm 164.70, 133.65, 133.47, 132.91, 132.62, 131.92, 128.39, 128.14, 126.63, 126.18, 126.02, 125.08 (2C), 63.82, 57.23 (2C), 53.98, 42.85, 42.65, 35.64, 27.80, 26.39, 26.23, 23.44, 21.19, 20.77; MS, m/z: 386.99 (M+). HRMS: calcd for $C_{26}H_{30}N_2O$ $(M+H)^+$, 386.2413; found, 387.2489.

SUPPLEMENTARY FIGURE AND TABLE

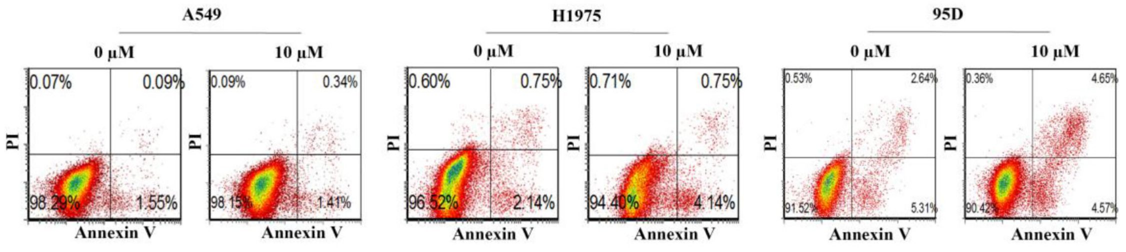

Supplementary Figure 1: YF-18 treatment could not induce cell apoptosis in A549, H1975, and 95D lung cancer cells.

Supplementary Table 1: Substrates of Skp2

Substrate	Function	Regulation
p27	Cell cycle inhibition	Skp2 induces the degradation of p27 [1]
E-cadherin	Cell migration regulation	Skp2 induces the degradation of E-cadherin [2]
p57	Cell cycle inhibition	Skp2 induces the degradation of p57 [3]
p21	Cell cycle inhibition	Skp2 induces the degradation of p21 [4]
p130	Cell cycle inhibition	Skp2 induces the degradation of p130 [5]
Smad4	Cell cycle arrest	Skp2 induces the degradation of cancer-derived Smad4 mutants[6]
CDK9	Transcriptional elongation	Skp2 triggers the degradation of CDK9 [7]
Myc	Cell cycle regulation and Apoptosis	Skp2 triggers the degradation of Myc [8]
FOXO1	Apoptosis	Skp2 triggers the degradation of FOXO1 [9]
MKP1	ERK signaling	Skp2 triggers the degradation of MKP1 [10]
TOB1	Cell cycle inhibition	Skp2 induces the degradation of TOB1 [11]
Brca-2	DNA repair	Skp2 triggers the degradation of Brca-2
CyclinD	Cell cycle regulation	Skp2 induces the degradation of CyclinD [4]
CyclinE	Cell cycle regulation	Skp2 induces the degradation of CyclinE [12]
Orc1p	DNA replication	Skp2 triggers the degradation of Orc1p [13]
E2F1	Cell cycle regulation/Apoptosis	Skp2 induces the degradation of E2F1 [14]
MEF	Cell cycle regulation	Skp2 induces the degradation of MEF [15]
Myb	Cell cycle regulation	Skp2 causes the degradation of Myb [16]
RASSF1A	Cell cycle regulation	Skp2 induces the degradation of RASSF1A [17]
Cdt1	DNA replication	Skp2 triggers the degradation of Cdt1 [18]
Rag-2	DNA recombination	Skp2 triggers the degradation of Rag-2 [19]
UBP43	Type 1 IFN signaling	Skp2 triggers the degradation of UBP43 [20]

REFERENCES

1. Tsvetkov LM, Yeh KH, Lee SJ, Sun H and Zhang H. p27(Kip1) ubiquitination and degradation is regulated by the SCF(Skp2) complex through phosphorylated Thr187 in p27. Curr Biol. 1999; 9:661-664.
2. Inuzuka H, Gao D, Finley LW, Yang W, Wan L, Fukushima H, Chin YR, Zhai B, Shaik S, Lau AW, Wang Z, Gygi SP, Nakayama K, Teruya-Feldstein J, Toker A, Haigis MC, et al. Acetylation-dependent regulation of Skp2 function. Cell. 2012; 150:179-193.
3. Kamura T, Hara T, Kotoshiba S, Yada M, Ishida N, Imaki H, Hatakeyama S, Nakayama K and Nakayama KI. Degradation of p57Kip2 mediated by SCFSkp2-dependent ubiquitylation. Proc Natl Acad Sci U S A. 2003; 100:10231-10236.
4. Yu ZK, Gervais JL and Zhang H. Human CUL-1 associates with the SKP1/SKP2 complex and regulates p21(CIP1/WAF1) and cyclin D proteins. Proc Natl Acad Sci U S A. 1998; 95:11324-11329.
5. Bhattacharya S, Garriga J, Calbo J, Yong T, Haines DS and Grana X. SKP2 associates with p130 and accelerates p130 ubiquitylation and degradation in human cells. Oncogene. 2003; 22:2443-2451.
6. Liang M, Liang YY, Wrighton K, Ungermannova D, Wang XP, Brunicardi FC, Liu X, Feng XH and Lin X. Ubiquitination and proteolysis of cancer-derived Smad4 mutants by SCFSkp2. Mol Cell Biol. 2004; 24:7524-7537.
7. Kiernan RE, Emiliani S, Nakayama K, Castro A, Labbe JC, Lorca T, Nakayama Ki K and Benkirane M. Interaction between cyclin T1 and SCF(SKP2) targets CDK9 for ubiquitination and degradation by the proteasome. Mol Cell Biol. 2001; 21:7956-7970.
8. von der Lehr N, Johansson S, Wu S, Bahram F, Castell A, Cetinkaya C, Hydbring P, Weidung I, Nakayama K, Nakayama KI, Soderberg O, Kerppola TK and Larsson LG. The F-box protein Skp2 participates in c-Myc proteosomal

degradation and acts as a cofactor for c-Myc-regulated transcription. Mol Cell. 2003; 11:1189-1200.

9. Huang H, Regan KM, Wang F, Wang D, Smith DI, van Deursen JM and Tindall DJ. Skp2 inhibits FOXO1 in tumor suppression through ubiquitin-mediated degradation. Proc Natl Acad Sci U S A. 2005; 102:1649-1654.
10. Lin YW and Yang JL. Cooperation of ERK and SCFSkp2 for MKP-1 destruction provides a positive feedback regulation of proliferating signaling. J Biol Chem. 2006; 281:915-926.
11. Hiramatsu Y, Kitagawa K, Suzuki T, Uchida C, Hattori T, Kikuchi H, Oda T, Hatakeyama S, Nakayama KI, Yamamoto T, Konno H and Kitagawa M. Degradation of Tob1 mediated by SCFSkp2-dependent ubiquitination. Cancer Res. 2006; 66:8477-8483.
12. Fujita T, Liu W, Doihara H and Wan Y. An *in vivo* study of Cdh1/APC in breast cancer formation. Int J Cancer. 2009; 125:826-836.
13. Mendez J, Zou-Yang XH, Kim SY, Hidaka M, Tansey WP and Stillman B. Human origin recognition complex large subunit is degraded by ubiquitin-mediated proteolysis after initiation of DNA replication. Mol Cell. 2002; 9:481-491.
14. Su S, Chen X, Geng J, Minges JT, Grossman G and Wilson EM. Melanoma antigen-A11 regulates substrate-specificity of Skp2-mediated protein degradation. Molecular and cellular endocrinology. 2016; 439:1-9.
15. Liu Y, Hedvat CV, Mao S, Zhu XH, Yao J, Nguyen H, Koff A and Nimer SD. The ETS protein MEF is regulated by phosphorylation-dependent proteolysis via the protein-ubiquitin ligase SCFSkp2. Mol Cell Biol. 2006; 26:3114-3123.
16. Kitagawa K, Kotake Y and Kitagawa M. Ubiquitin-mediated control of oncogene and tumor suppressor gene products. Cancer Sci. 2009; 100:1374-1381.
17. Song MS, Song SJ, Kim SJ, Nakayama K, Nakayama KI and Lim DS. Skp2 regulates the antiproliferative function of the tumor suppressor RASSF1A via ubiquitin-mediated degradation at the G1-S transition. Oncogene. 2008; 27:3176-3185.
18. Li X, Zhao Q, Liao R, Sun P and Wu X. The SCF(Skp2) ubiquitin ligase complex interacts with the human replication licensing factor Cdt1 and regulates Cdt1 degradation. J Biol Chem. 2003; 278:30854-30858.
19. Jiang H, Chang FC, Ross AE, Lee J, Nakayama K, Nakayama K and Desiderio S. Ubiquitylation of RAG-2 by Skp2-SCF links destruction of the V(D)J recombinase to the cell cycle. Mol Cell. 2005; 18:699-709.
20. Tokarz S, Berset C, La Rue J, Friedman K, Nakayama K, Nakayama K, Zhang DE and Lanker S. The ISG15 isopeptidase UBP43 is regulated by proteolysis via the SCFSkp2 ubiquitin ligase. J Biol Chem. 2004; 279:46424-46430.
